# Gray's Impulsivity Is Differentially Associated With Amygdala-Insula Functional Connectivity in Adolescents, Depending on ADHD Risk Status

**DOI:** 10.1192/bjo.2022.262

**Published:** 2022-06-20

**Authors:** Kristóf Ágrez, Nóra Bunford

**Affiliations:** Developmental and Translational Neuroscience Research Group, Institute of Cognitive Neuroscience and Psychology, Research Centre for Natural Sciences, Budapest, Hungary

## Abstract

**Aims:**

Attention-Deficit/Hyperactivity Disorder (ADHD) is associated with alterations in both reinforcement sensitivity and affective processing but the nature of the associations of these characteristics is yet to be examined. We hypothesized that individual differences in the sensitivity of the Behavioral Approach System (BAS) would exhibit differential relations with affective network connectivity – involved in emotional regulation and salience monitoring – in youth at-risk for, relative to youth not at-risk for, ADHD.

**Methods:**

Adolescents (*n* = 125; *M*_age_=16.24 years, *SD* = 1.09 years; 61.6% boys) were recruited as part of The Budapest Longitudinal Study of ADHD and Externalizing Disorders. Forty-nine were classified as at-risk for ADHD (*M*_age_=16.15 years; *SD* = 1.21 years; 77.6% boys), defined as exhibiting ≥4 parent-rated symptoms of either domain on the ADHD Rating Scale-5. Participants completed a 10-minute resting-state functional Magnetic Resonance Imaging session, during which they were asked to focus their attention on a fixation cross, as well as various self-report assessments, including the Reinforcement Sensitivity Theory of Personality Questionnaire (RST-PQ).

**Results:**

Functional Network Connectivity analyses indicated an interaction effect between the RST-PQ BAS impulsivity subscale and at-risk status on functional connectivity between four affective network region-pairs (*p*s < .05, False Discovery Rate [FDR] corrected) within a cluster based on functional similarity (*p* = .014, FDR-corrected). Follow-up OLS linear regressions showed higher impulsivity scores predicted stronger functional connectivity between the (1) left amygdala-right insula (*F*(6, 117) = 3.298, *p* = .005, adjusted *R*^2^=.101), (2) left amygdala-left insula (*F*(6, 117) = 2.2, *p* = .048, adjusted *R*^2^=.055), (3) right amygdala-right insula (*F*(6, 117) = 3.833, *p* = .002, adjusted *R*^2^=.121), and (4) right amygdala-left insula (*F*(6, 117) = 3.064, *p* = .008, adjusted *R*^2^=.092) in at-risk youth, whereas an inverse relationship was apparent in not at-risk youth.

There was no main effect of group status on BAS impulsivity scores (*t*(122)=−1.167, *p* = .246) or on functional connectivity ((1) *t*(122) = .383, *p* = .702; (2) *t*(122) = .195, *p* = .846; (3) *t*(122)=−.107, *p* = .915; (4) *t*(122)=−.206, *p* = .837).

**Conclusion:**

The amygdala-insula connection has been shown to be involved in trait impulsivity, however, available ADHD-focused studies targeted emotional functioning. To our knowledge, we are the first to demonstrate that Gray's impulsivity – reflecting trait reward sensitivity – is predictive of amygdala-insula functional connectivity at rest and that this relation differs given ADHD risk. Results have conceptual and practical implications (e.g., early identification) as the role of the amygdala-insula connection in reward sensitivity appears especially relevant for a developmental phase and a diagnostic group linked to increased risk taking.

